# Attitudes of medical students towards psychiatry and mental illnesses: a cross-sectional study from Pakistan

**DOI:** 10.1192/bjo.2021.802

**Published:** 2021-06-18

**Authors:** Syed Muhammad Jawad Zaidi, Muhammad Hamza, Raja Adnan Ahmed, Mishal Fatima, Hassan Nadeem, Mehwish Kaneez

**Affiliations:** 1 Rawalpindi Medical University; 2 Consultant Psychiatrist, Cmw Taf Morhannwg University Health board; 3 Final Year MBBS student, Rawalpindi Medical University; 4 Jinnah Sindh Medical Univeristy, Dudley group NHS Foundation Trust

## Abstract

**Aims:**

The increasing burden of mental disorders coupled with the social stigmatization in Pakistan is an immense barrier in combating the emerging mental health crisis. The low number of qualified psychiatrists and poor intake in post-graduate psychiatry training programs in the region further complicates the problem. Thus, our study aims to assess the attitudes of Pakistani medical students towards psychiatry. Furthermore, we also aim to evaluate how experience and different levels of exposure to psychiatry among students affect their attitudes towards psychiatry as a career choice.

**Method:**

This cross-sectional study was conducted via an online survey made on Google Forms. A total of 831 medical students studying across various private and public medical institutions of Pakistan responded to the survey. The questionnaire comprised of demographical details (gender, age, institution, and academic year) exposure to psychiatry, duration of psychiatry rotation, and personal experience with mental illness. The attitudes of medical students towards psychiatry were evaluated using the English version of the 30-item Attitudes Towards Psychiatry (ATP-30) scale. Chi-square test and multiple regression with backward method were used to analyze the data.

**Result:**

The Cronbach's alpha value of the ATP-30 scale was 0.830. The participants in our study had a mean score of 107.6 ± 12 on ATP-30. Overall, most participants had a positive attitude towards psychiatry. Multiple regression analysis revealed a significant model pertaining to predictors of attitude toward psychiatry (F (df) = 11.28 (830), P < 0.001). However, the predictors included in the model accounted for only 5.8% of the variation in ATP-30 scores. According to it, those students had a more positive attitude toward psychiatry who identified as female, older and having any sort of exposure toward psychiatric specialty, direct involvement in psychiatric patient care, and reporting personal experience of mental illnesses.

**Conclusion:**

Our study showed that medical students had a positive attitude towards psychiatry but female medical students, students with previous exposure to psychiatry, and students with longer psychiatry rotations tend to view psychiatry more positively. The generally positive trend towards psychiatry in Pakistan indicates the need to sustain improvements through proactive measures. We recommend longer placements for medical students in mental health settings for at least 4 weeks or longer. Medical schools should also promote research, discussions, and seminars on different psychiatric illnesses in order to enhance awareness among the students.

